# Volume rendering technique and high-resolution microCT: 3D exploration of the cochlear anatomy

**DOI:** 10.1007/s00405-025-09360-6

**Published:** 2025-04-03

**Authors:** Gabriela O’Toole Bom Braga, Robert Zboray, Annapaola Parrilli, Franca Wagner

**Affiliations:** 1 Artorg Center for Biomedical Engineering Research, University of Bern, Bern, Switzerland; 2 Center for X- Ray Analytics, Empa, Swiss Federal Laboratories for Materials Science and Technology, Dübendorf, Switzerland; 3 Department of Diagnostic and Interventional Neuroradiology Institute of Diagnostic and Interventional Neuroradiology, Inselspital, Bern University Hospital, University of Bern, 3010 Bern, Switzerland; 4 Department of Otorhinolaryngology - Head and Neck Surgery, Inselspital, University Hospital University of Bern, University of Bern, Bern, Switzerland

**Keywords:** MicroCT, Cochlea, Volume rendering, Anatomy

## Abstract

**Purpose:**

Given its unique anatomical position and the amalgamation of bony and soft tissues within the cochlea, exploring its intricacies poses persistent challenges. Histopathology remains the gold standard in research, but given its inherent limitations, there is a clear need for innovative alternatives. The integration of microCT technology with advanced volume rendering techniques emerges as a promising approach for overcoming the hurdles associated with anatomical investigations of the cochlea.

**Methods:**

We seamlessly integrated high-resolution microCT cochlear images with medical imaging analysis software to create detailed 3D anatomical images of the human cochlea without the need of sample processing.

**Results:**

Volume rendering allowed a multiplanar, non-destructive, detailed anatomical evaluation of the human cochlea, including its capillary system, as well as soft tissue visualization at single-micron resolution in 3D.

**Conclusion:**

The use of volume rendering in cochlear anatomical studies is underexplored despite the prevalence of 3D reconstruction. This technique presents a promising avenue for scientific investigation, providing researchers with unprecedented insights that can potentially benefit patients with hearing disorders.

## Introduction

Deciphering the cochlea anatomy has been a long-time challenge due to its encasement on the hardest bone of the human skull, the petrous bone. Although histopathology remains the gold standard, tissue preparation, artifact introduction, time, costs, and anisotropic resolution, can potentially interfere on the final outcome [1]. Alternatively, laboratory-based micro-computed tomography (microCT) technology has been able to demonstrate previously unseen details of the human cochlea anatomy without introducing artifacts or destroying the sample, while adding a third dimension that fails to manifest on conventional techniques [1–4].

Routine methodology in histopathological studies uses fixation, decalcification, infiltration, and dehydration, as a preparation for serial-sectioning [4–6]. As consequence, the procedure becomes labor intensive, can last several weeks up to months, cause tissue shrinkage (when calcium concentrations are decreased) and distortion/swelling (when calcium concentrations are increased) of the examined organs [6]. To understand the dynamic interactions among the cochlear components and the entangled anatomy of the cochlea partition (CP), it is imperative that the inner cochlear elements have not been altered during tissue preparation [3, 5, 6].

Regarding 3D imaging, conventional histology presents considerable limitations. The destructive and time-consuming methodology used to record the entire organ or large field of views (FOVs) by parallel sections, make it near impossible to cover the complete cytoarchitecture of the organ [7]. By contrast, microCT is able to assess cochlea tissues and allow for 3D reconstructions with selectable FOV at single and even nano-micron ranges. MicroCT also allows for concomitant analysis of the entire organ or limited areas covering a region of interest (ROI). Voxel sizes can be chosen from the micro to the nano range, when high resolution detectors and/or magnification in cone-beam geometries are used [7].

Albeit the needed improvement in staining techniques and imaging resolution to visualize more cytological details, microCT anatomical studies can already provide state-of-the-art 3D reconstructions and pristine anatomical descriptions of the human cochlea [1, 2]. Alternatively, the use of medical software to apply segmentation techniques can also improve the visualization of the cochlea anatomy, including soft tissue [8]. Through the use of imaging software, it is possible to reconstruct anatomy, highlight specific tissues and virtually remove artifacts or anatomical structures without compromising the final outcome or specimen integrity [1, 2].

Here, we associate microCT imaging with volume rendering techniques creating detailed 3D images of the inner ear to push the boundaries of cochlear anatomical studies. By encompassing surgical techniques, single-micron explorations, multiplane evaluations with volume rendering techniques, we aim to provide a comprehensive and detailed 3D understanding of the human cochlea at microscopic level unraveling the intricacies of the cochlea's architecture.

## Materials and methods

### Image data

With the approval of the local ethics review board (KEK Bern, Switzerland, Project-ID 2018-00770), two cadaveric samples of formalin-flushed human cochleae were analyzed in this study. Specimens were chosen randomly from the whole head specimens available, and the temporal bones were carefully removed (two right sides).

### Cochlea removal

Owing to the limitations of the detector size and to avoid excessive harmful absorption by the surrounding bone structures, the temporal bone surrounding the cochlea had to be removed. The dissection started with middle ear inspection. The two cochleae showed no signs of previous surgical procedures or middle ear disease. The post-auricular incision was made, and the mastoid bone was exposed. Later, a mastoidectomy and subsequent labyrinthectomy, as described by Gulya was performed, followed by cochlear refinement until de organ was no bigger than 1.5 cm [1, 2, 9].

### MicroCT

Prior to the scanning, the cochleae were kept in iodine solution for 3 days, without opening the round and oval window membranes. Therefore, counting only on the diffusion principle for tissue staining.

MicroCT analysis was carried out using an EasyTom XL Ultra 230–160 micro/nanoCT scanner (RX Solutions, Chayanod, France). The scanner features a Hamamatsu nano-focus, transmission X-ray tube with a 1 µm-thick tungsten target on a diamond window. The tube was operated with an LaB6 cathode. The scans were performed using a Varian PaxScan 2520DX detector (flat panel with amorphous silicon and a CsI conversion screen; 1920 × 1536-pixel matrix; pixel pitch of 127 µm^2^; 16 bits of dynamic range). The tube was operated at 70 kV and a current of 60 µA. The full cochlea sample was scanned at a voxel size of 13 and 14 µm (cochleae 1 and 2, respectively). The overall size of the samples harvested for microCT analysis is slightly different both because of normal variability between samples and because of the surgical refinement during the preparation procedure (dissection after removal). Additional zoom scans were performed in order to not physically cut the sample but to allow a more detailed reconstruction of the internal structures with different voxel sizes (2.3 μm, to 2.5 μm depending on the available FOV).

### 3D reconstructions of the cochlea anatomy

For the 3D reconstructions of the cochlea soft tissue, cochlea bone and capillary systems, the medical image analysis software Amira (Thermo Fisher Scientific, Waltham, Massachusetts, USA), was used. For the full cochlea and the basal and mid turns, we segmented the datasets using global thresholds to create the 3D images. The bone was segmented, and the selected voxels were added to a label; any noise was removed using the brush tool. This label was later multiplied by the original.tiff image to isolate the labeled anatomy and, using the volume rendering tool, the 3D mesh image was created. Using additional thresholding, the soft tissue was segmented, and the selected voxels were added. In this case the noise was not removed, as it corresponded to the soft tissue seen on the raw image.

## Results

**Fig. 1:**
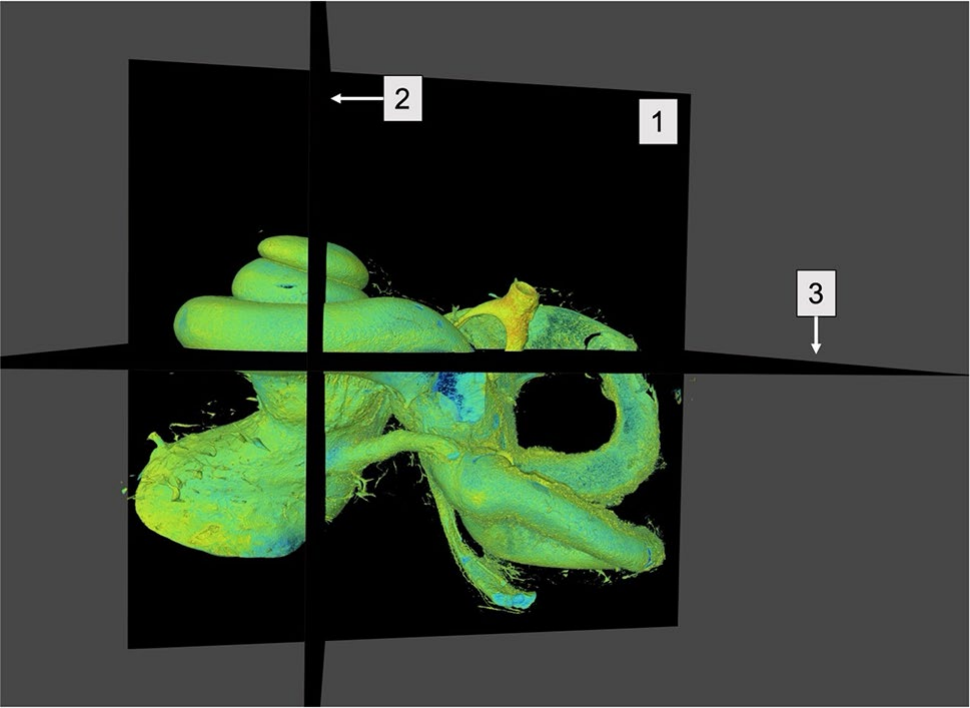
Considering this anatomical view of the cochlea, using microCT, multiplane reconstructions are now possible. **A** Multiplane reconstruction using microCT; **1** Sagittal plane; **2** Coronal plane; **3** Axial plane

We were able to demonstrate that is possible to visualize structural anatomy in a multiplane analysis (Fig. 1) using microCT technology and volume rendering techniques, without the need of sample preparation. MicroCT was also used to create 3D reconstructions of the cochlea anatomy, through the use of mathematical calculations that revealed to be a new methodology for anatomical studies of the human cochlea. It is relevant to mention that this type of reconstructions also permits detailed quantitative study [1, 2]. Here we intend to demonstrate that mathematical calculations used on imaging medical software to create 3D images also allows a thorough examination of the cochlea structures under microCT.

**Fig. 2:**
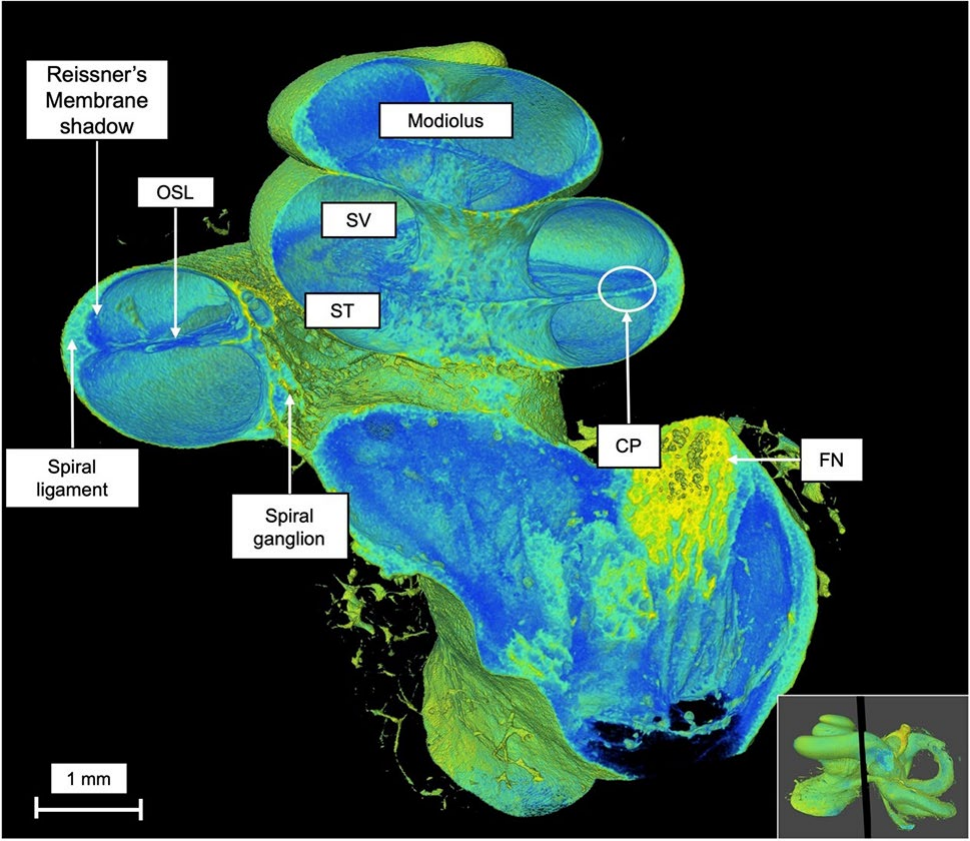
Coronal 3D reconstruction of the human cochlea 14 µm voxel size. *OSL* osseous spiral lamina, *SV* scala vestibuli, *ST* scala tympani, *CP* cochlear partition; FN- facial nerve

**Fig. 3:**
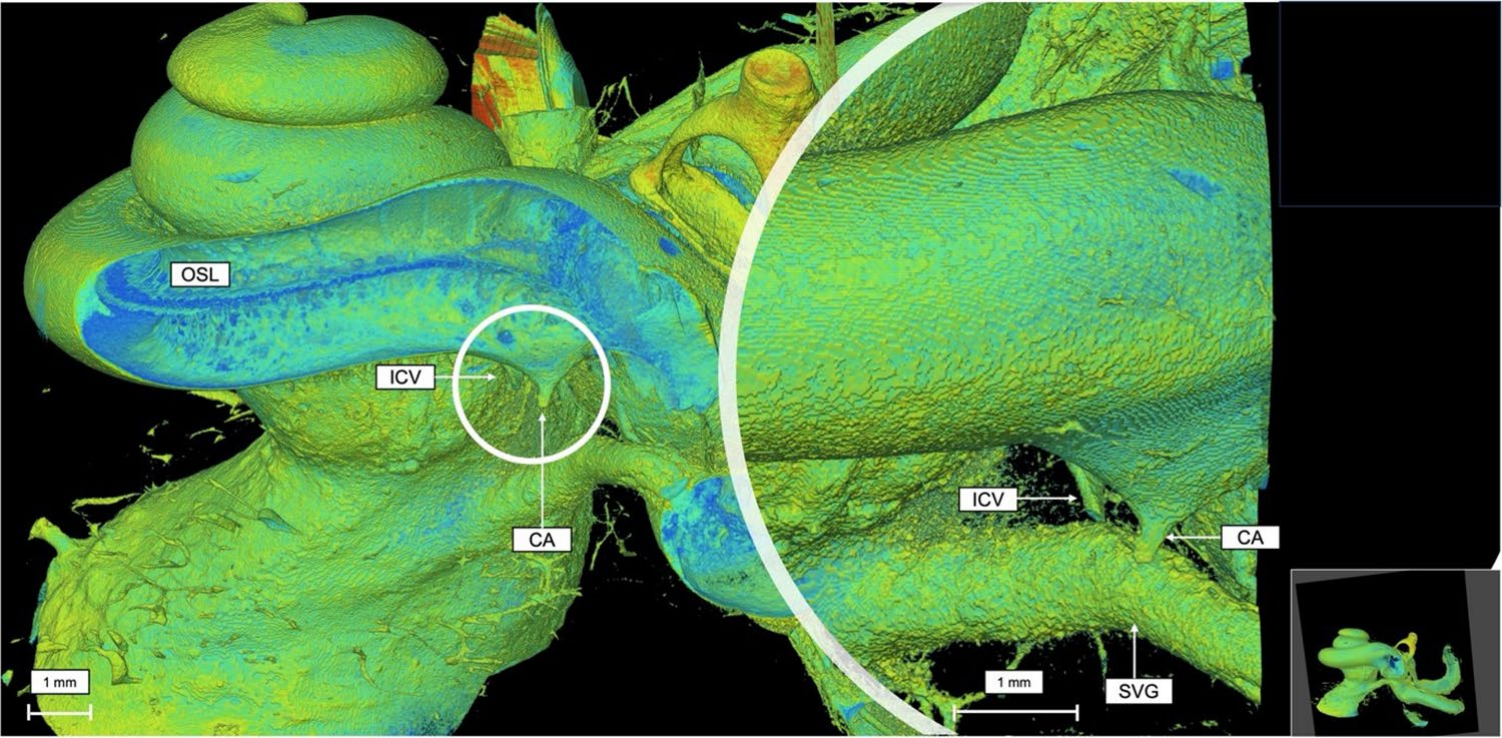
Sagittal view of the cochlea at 14 µm voxel size, showing the inferior cochlear vein and the cochlear aqueduct in detail. *OSL* osseous spiral lamina, *ICV* inferior cochlear vein, *CA* cochlear aqueduct, *SVG* superior vestibular ganglion

**Fig. 4:**
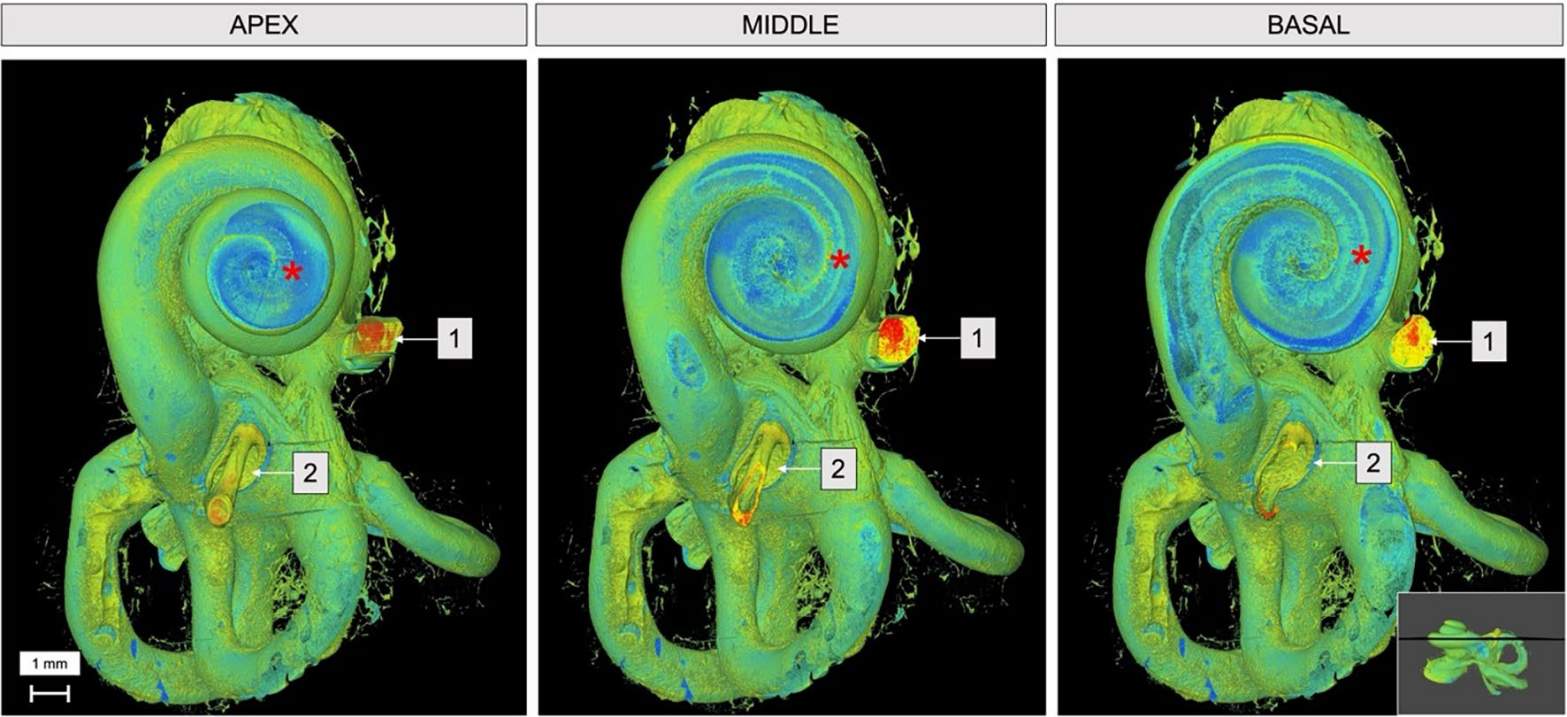
Simultaneous axial plane reconstruction at 14 µm voxel size of the cochlea apex, middle and basal turns. **1** Facial nerve; **2** Stapes; Red star-OSL

A coronal virtual dissection at 14 μm voxel size of the human cochlea (Fig. 2), permitted a detailed visualization of most of the cochlea structures in all spirals and the internal auditory canal (IAC). Including: the spiral ligament (SL), spiral ganglion (SG), the osseous spiral lamina (OSL), shadow view of the Reissner’s membrane, scala tympani and vestibuli (SV and ST), the modiolus, cochlear partition (CP) and the facial nerve (FN). By generating a sagittal view of the cochlea (Fig. 3), it is possible to observe the cochlear aqueduct (CA) and the inferior cochlear vein (ICV), as well as the superior vestibular ganglion (SVG) while entering the vestibule. MicroCT also allows a multislice axial reconstruction on the axial plane, granting simultaneous view of the apex, middle turn, and basal turn (Fig. 4).

**Fig. 5:**
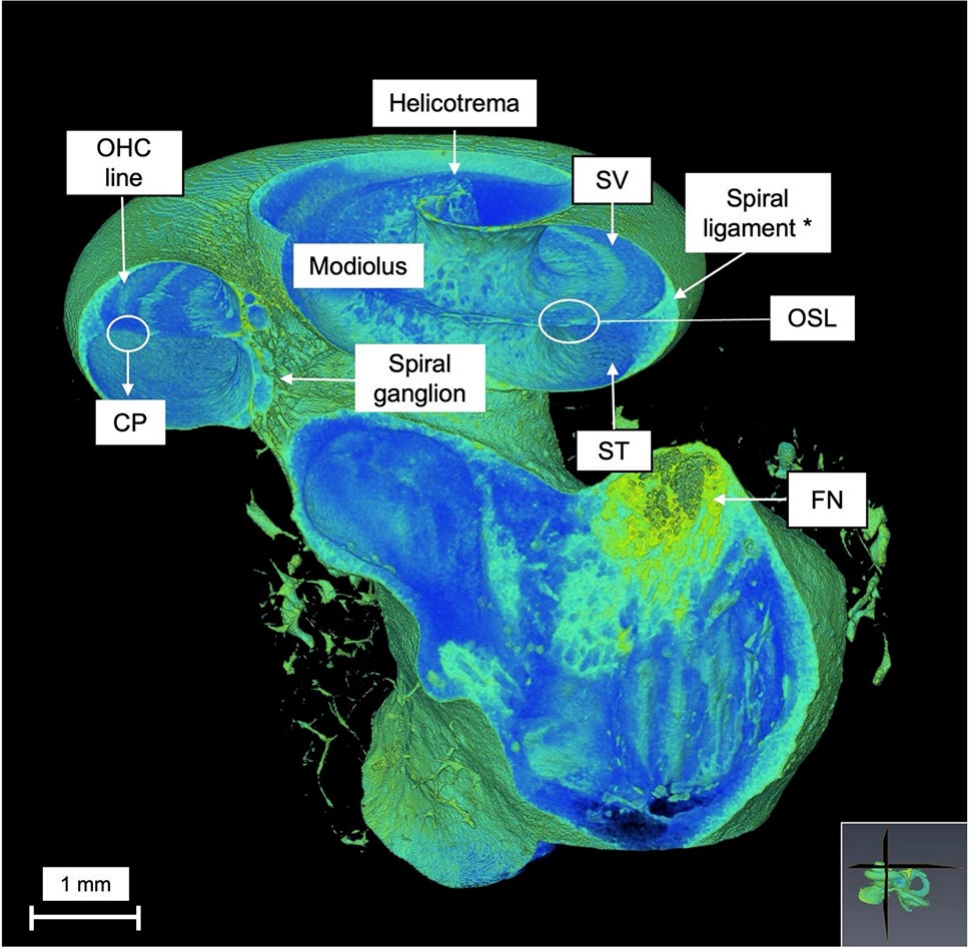
Multiplane reconstruction (coronal and axial) of the cochlea at 14 µm voxel size resolution. (*) Spiral ligament. Note that the 3D reconstruction of a non-decalcified, 3-days-iodine-stained cochlea allow of the observation of the outer hair cells (OHC) line

Using a multiplane slice on both coronal and axial planes (Fig. 5), it is possible to analyze, at the same time, the basal, middle turns, and the apex of the cochlea. Making it possible to visualize, at the same time the helicotrema, the CP, modiolus, SL, the OSL, SV and ST.

**Fig. 6:**
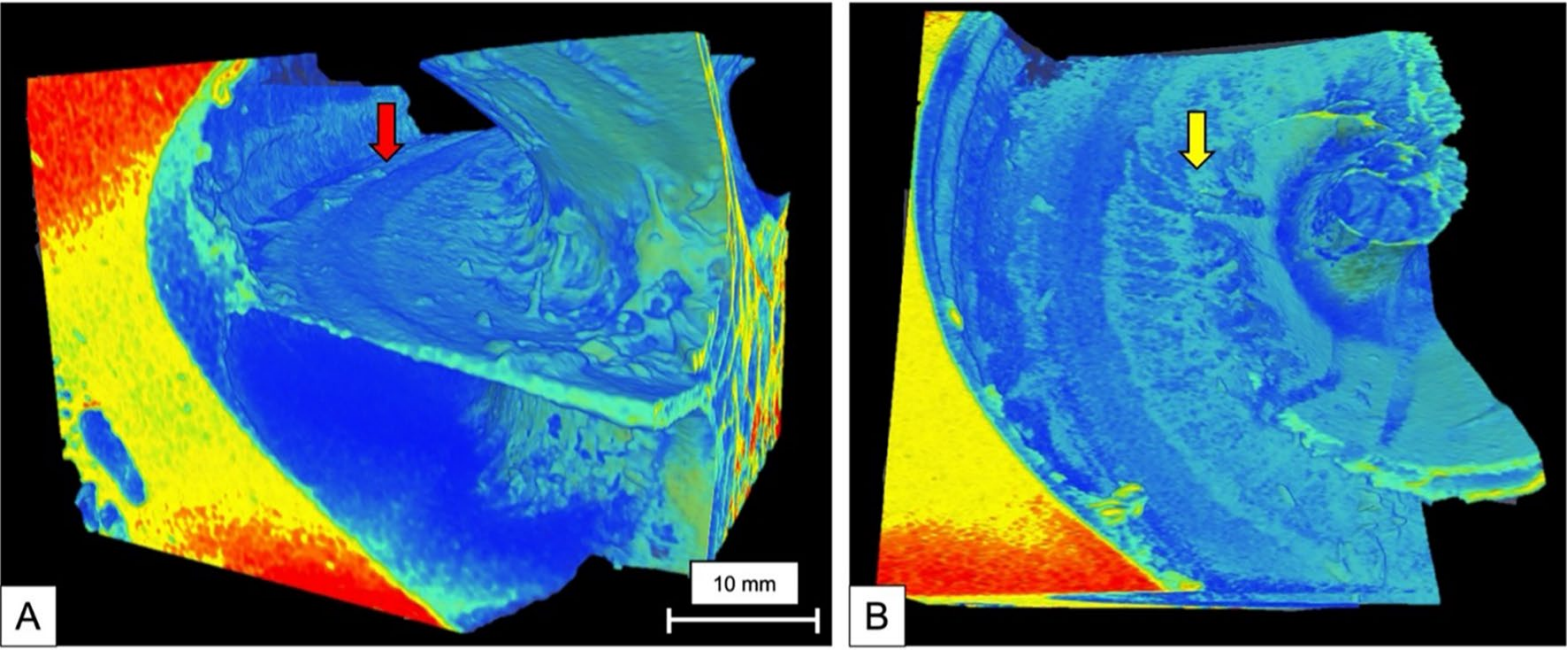
3D mesh reconstruction of the basal turn of the cochlea at 2.3 µm voxel size. Using a different colormap, it was possible to demonstrate: **A** the roll of the outer hair cells on the cochlear partition (red arrow); and **B** the osseous spiral lamina (yellow arrow)

**Fig. 7:**
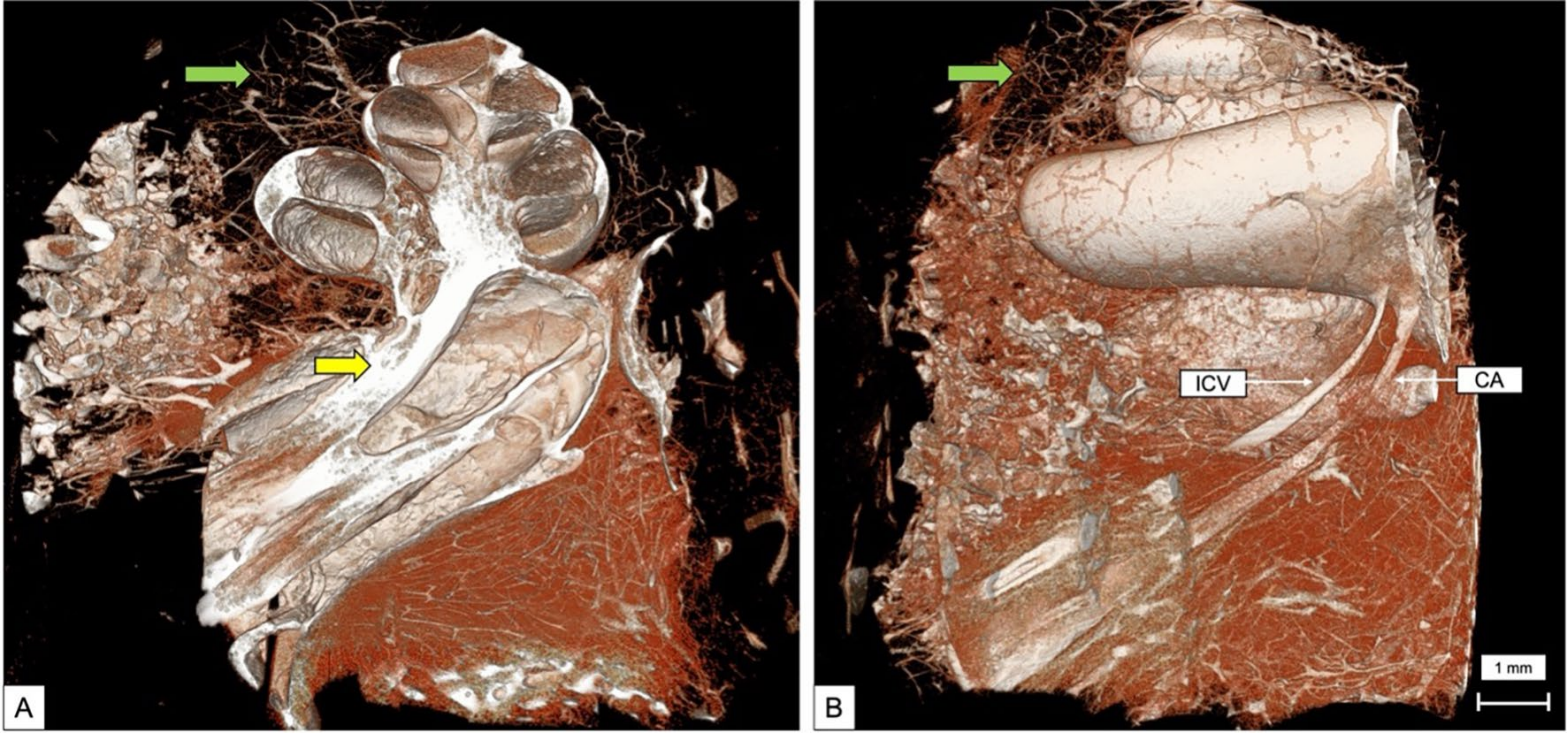
**A** 3D reconstruction of the cochlea vascular system. Notice the increased vascularity surrounding the internal auditory canal and the cochlear nerve (yellow arrow), and the apex (green arrow). **B** Sagittal view of the vascular capillaries on the apex (green arrow) and the vessels surrounding the ICV and CA. *ICV* inferior cochlear vein, *CA* cochlear aqueduct

Applying a similar colormap that was used on full cochlea reconstructions to the 2.3 µm raw image of the basal turn, it was possible to closely examine the anatomy of the cochlea partition (Fig. 6). On Fig. 6A the roll of the outer hair cells (OHC) can be seen (red arrow), while on Fig. 6B, it is possible to identify the OSL (yellow arrow). Visualizing the capillary vascular system surrounding the cochlea, was possible using a different colormap on the 3-day- stained cochlea (Fig. 7). Additionally, with this colormap it is possible to notice the cochlear nerve (yellow arrow) entering the internal auditory canal.

**Fig. 8:**
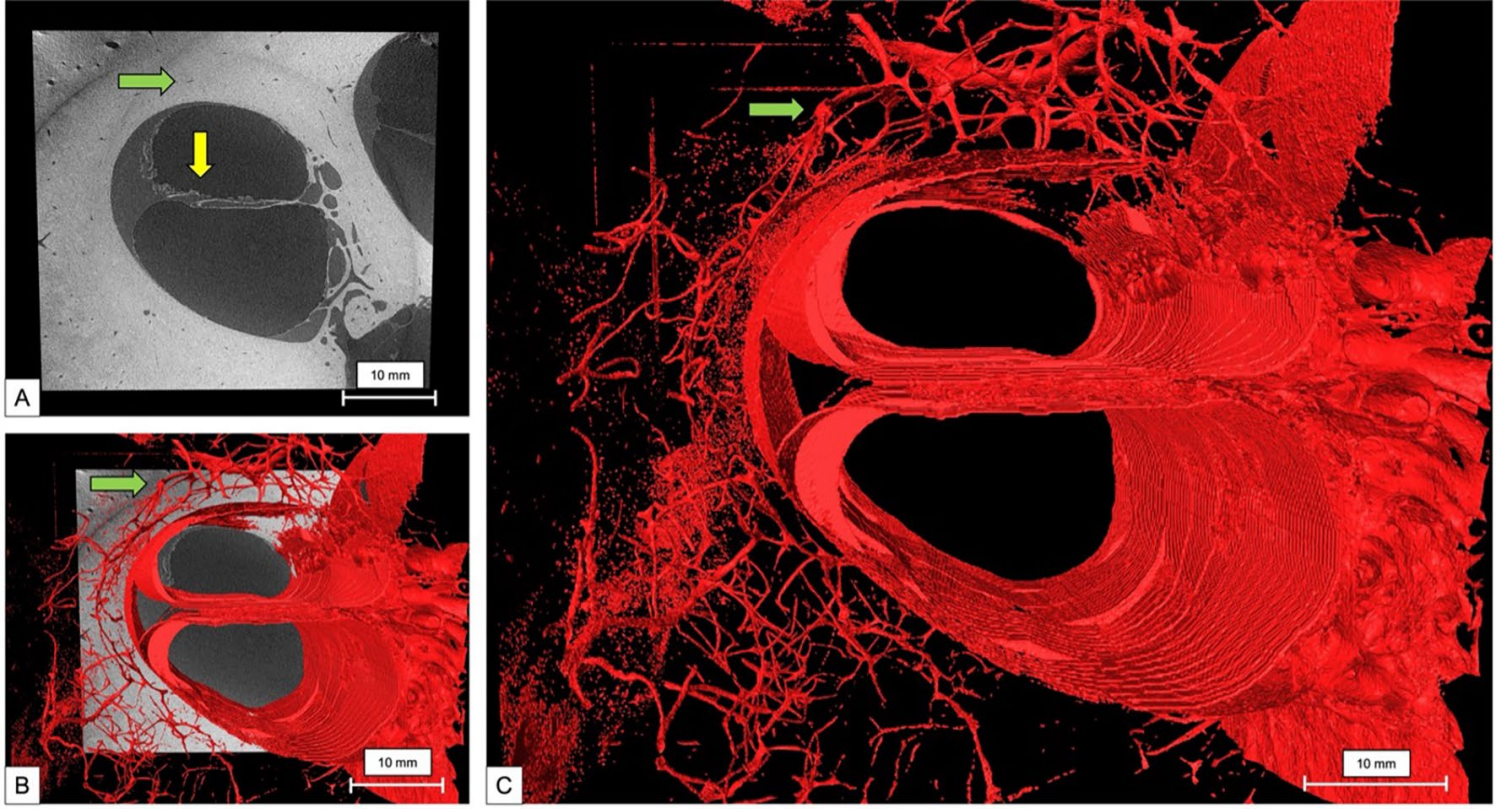
**A** Raw image of the human cochlea at 2.3 µm voxel size. Yellow arrow shows the Bridge region on the human cochlea. Green arrow demonstrates the bony canals of the capillary vasculature of the cochlea. **B** Raw image and 3D reconstruction of the vascular capillary system. **C** 3D mesh reconstruction of the human cochlea at 2.3 µm voxel size, with emphasis on the capillary system surrounding the cochlea

Using 2.3 µm nominal resolution, it was possible to evaluate the basal turn vascular system and the specific anatomy of the cochlea partition in humans (Fig. 8). The Bridge region can be observed where the CP attaches to the OSL (Fig. 8A-yellow arrow). Green arrow on 8A, displays the tunnels where the capillaries are found. By adding the 3D reconstruction to the raw image, it is possible to recognize the vessels emerging from the raw image tunnels (Fig. 8B-green arrow). Figure 8C displays the vascular 3D reconstruction at single-micron resolution of the basal turn of the cochlea.

## Discussion

Investigating cochlear morphology is important from numerous points of view, including sensory physiology and cochlear modeling, as well as to follow anatomical developmental changes, disease related transformation and genetic diseases. However, “classical” cochlear studies remain challenging for most imaging methods require specimen preparation, destruction, and lack spatial resolution. Even when using alternative techniques to histopathology such as the hemi-cochlea to study fresh and unfixed cochlear tissue, distortion of the cochlear membranes is still expected [5, 6, 10]. In addition, histopathology creates 2D data, therefore providing less information than microCT (3D). For example, a higher OSL porosity was recently described in a study that used microCT information as data source when compared to other study that used histological cuts to create 3D images as a method [1, 11]. Also, porosity of the individual OSL plates could be quantified, while bony pillars between the OSL were visualized in detail providing valuable information to be added to future anatomical and mathematical models used in hearing kinetic studies [1].

The cochlea is particularly difficult for imaging analysis due to its encasement on the petrous bone and the fact that it consists of a blend of bony and soft tissue. In research, high resolution X-Ray imaging is an option to overcome the aforementioned obstacles while providing micrometer resolution and allowing for 3D mesh reconstructions. Although the absorption of X-ray by soft tissues is weak, dynamic studies of the cochlea are feasible using image analysis software that allow for 3D reconstructions by using threshold adjustments and volume rendering. Multi-Planar Reconstruction (MPR) can be used on microCT generated images providing new valuable anatomical information at single-micron resolution [3, 4]. Volume rendering and specific colormaps can be used together with MPR to further improve the anatomical understanding. Colormaps make it possible to highlight certain structures while virtually suppressing others by granting different colors according to the tissue gradient. For example, on the colormap used to demonstrate the vascularity of the cochlea we added the red color to the low gradient region while suppressing higher contrast areas. By using threshold adjustments between the different density tissues, it was possible to mark the gradient of interest and recreate the capillary system surrounding the cochlea.

Coronal plane analysis for the 14 µm voxel sized cochlea made it possible to concomitantly assess all 2 and a half cochlear spirals. Combining manual and semi-automatic segmentation methods in addition to imaging multiplication (segmented x original.tiff image), it was possible to recreate the soft tissue in 3 dimensions. The segmentation process can take a few minutes to complete when the operator is familiar with the software and its tools. Since the image sizes are big (up to 1 T/cochlea), it is important to have computer power to make the process even faster. Once the process is complete, volume renderings of the cochlea soft tissue were displayed and could be manipulated in the orthogonal planes of the scanned volume showing the cochlear partition, spiral ligament, the facial nerve, as well as bony structures such as the osseous spiral lamina, modiolus and the spiral ganglion. Despite the use of 3D imaging generation not being a novelty for ear research, it is still not common to use volume rendering to demonstrate the complicated local anatomy [3, 4]. For example, the FN fibers could be seen in its full entering the labyrinthine segment and making up the first segment of the fallopian canal.

Similarly, the sagittal plane reconstruction made it feasible to identify on the same image the inferior cochlear vein, cochlear aqueduct, superior vestibular ganglion, the osseous spiral lamina and perform a full anatomical study of the local nerves. Imaging segmentation, as described by Braun et al. [4], is the generation of images of connected regions by integration, originating surfaces that allows quantitative volume determination. For example, we selected points related to the vestibulocochlear nerve (CN VIII) until it connected to the vestibule. These points were added to a material e.g. nerve, and a colormap was created highlighting the nerve fibers and its trajectory. This same method was used to segment each and every anatomical structure herein analyzed. Adding to the segmentation, multiplane reconstruction in high resolution made the important anatomical relation between the inferior cochlear vein and the cochlear aqueduct clear to visualize. By offering the possibility to add or remove anatomical structures and to observe the organ from different angles, high resolution 3D segmentation of the cochlea can enhance the understanding of this complex anatomical organ.

Although soft tissue details can be lost while using microCT when certain preparation techniques are applied to the specimen, volume rendering tool allowed for a first sighting of the line of external hair cells [3, 4]. It is important to notice that microCT alone (raw image) doesn’t provide such anatomical details, therefore, it is imperative that volume rendering reconstructions are performed in order to increase soft tissue observation [12, 13]. Some authors advocate the use of osmium tetroxide (O_S_O_4_) as staining to enhance soft tissue contrast, despite costs and soft tissue effects [4, 14, 15]. In our study, one of our samples was submerged in iodine solution for three days, enhancing the contrast between the different density tissues, making segmentation through threshold adjustment easier to accomplish. It is important to mention that slice thickness ultimately determines the ability to define finer details [12]. Using single-micron resolution (2.3 µm voxel size) on the basal turn, combined with iodine staining, multiply by image techniques for surface generation, it was possible to observe the line of external hair cells of the CP in the finest detail. Fine surface details could be seen clearly despite the alternation in soft tissue and bone density characteristic from the organ.

While the application of 3D reconstruction techniques has become a well-established method in various fields, the incorporation of volume rendering in cochlear anatomical studies remains an area where widespread adoption is not yet commonplace. Despite the maturity of 3D reconstruction technologies, their integration into the specific domain of cochlear research presents a unique and underexplored frontier [12, 13]. Some advantages of the presented technique over current available methods lie on the fact that no sample preparation is needed. Postnov et al. [3], used an interesting preparation technique for one of their samples with the removal of the cochlea’s perilymph and decalcification on another, to reduce imaging artifacts. Although some of the bigger structures (i.e. SL) were well seen, details of the OSL, for example, were lost specially on the decalcified sample [4]. We believe that the local anatomy will be better preserved when no preparation whatsoever is used before the scanning. Although decalcification can produce an “ideal visualization” with transparency caused by the calcium removal, it can already cause intense tissue deformation [10]. Another advantage of our technique is that it allows for the seamless integration of spatial information, enabling researchers to visualize the cochlea in its entirety and gain a more comprehensive understanding of its structural detailed subtleties. Moreover, volume rendering techniques do not require great knowledge from the software operator to be implemented. This way even students or medical personnel that are unfamiliar with the software can be trained to use the system in little time and create 3D images. Nonetheless, for someone that is familiar with the software and has the necessary computer power (i.e. graphics card), the segmentation process and imaging creation can take only a few minutes making the procedure easy to reproduce. While availability of microCT scanners and scanning time can still be a challenge with great potential for improvement, the anatomical details presented on the 3D models make the technique worth exploring for otological research.

In addition, the use of dual resolutions (14 and 2.3 µm) made it possible for detailed reconstruction of the cochlea’s capillary system. At the higher resolution of 2.3 µm, fine details of the capillary network were unveiled with clarity, enabling a meticulous examination of the vascular intricacies surrounding the basal turn. In the case of the inferior cochlear vein, a feature of paramount importance in cochlear studies, its relationship with the cochlear aqueduct and the oval window was significantly enhanced through the application of this technique increasing the understanding of the vascular dynamics within the cochlea [7, 12, 16]. This high resolution was also able to recognize the Bridge junction described by Raufer [17] and its unique insertion to the OSL in humans. Our raw microCT image clearly shows the encounter of the soft tissue of the cochlear partition with the bony skeleton of the OSL.

Despite the existing wealth of knowledge about the human cochlea derived from conventional 2D imaging and 3D reconstruction methods, volume rendering offers a more comprehensive perspective. Even though some abilities such as surgical dexterity for the cochlea removal, availability of microCT scanning, computer power and software availability are essential to implement this technique, its quantifiable results and its impact in anatomical research compensate the efforts. Furthermore, the application of volume rendering in cochlear studies has the potential to facilitate interdisciplinary collaborations by providing vivid, intuitive visualizations that can be easily interpreted by experts from various fields. This can foster a more integrated approach to cochlear research, bringing together insights from bioengineers, anatomists, (neuro-)radiologists, and otolaryngologists.

## Conclusion

While 3D reconstruction has become a standard tool in scientific investigations, the untapped potential of volume rendering in cochlear anatomical studies represents a promising frontier. Associating surgical refinement, non-destructive staining techniques, microCT and imaging analysis, can elevate our understanding of cochlear structures to new heights offering researchers unprecedented anatomical insights with quantifiable capability that will further the knowledge in hearing studies.

## Data Availability

The raw data supporting the conclusions of this article will be made available by the authors, without undue reservation.

## References

[1] Bom Braga GO et al (2023) Quantitative Evaluation of the 3D Anatomy of the Human Osseous Spiral Lamina Using MicroCT. J Assoc Res Otolaryngol 24(4):441–452 37407801 10.1007/s10162-023-00904-3 PMC10504225

[2] O’Toole Bom Braga G, Zboray R, Parrilli A, Bulatovic M, Caversaccio MD, Wagner F (2022) Otosclerosis under microCT: New insights into the disease and its anatomy. Front Radiol 2:965474. 10.3389/fradi.2022.965474 37492684 10.3389/fradi.2022.965474 PMC10365283

[3] Postnov A et al (2006) High resolution micro-CT scanning as an innovative tool for evaluation of the surgical positioning of cochlear implant electrodes. Acta Otolaryngol 126(5):467–474 16698695 10.1080/00016480500437377

[4] Braun K, Bohnke F, Stark T (2012) Three-dimensional representation of the human cochlea using micro-computed tomography data: presenting an anatomical model for further numerical calculations. Acta Otolaryngol 132(6):603–613 22384791 10.3109/00016489.2011.653670

[5] Rau C, Robinson IK, Richter CP (2006) Visualizing soft tissue in the mammalian cochlea with coherent hard X-rays. Microsc Res Tech 69(8):660–665 16788978 10.1002/jemt.20336

[6] Edge RM et al (1998) Morphology of the unfixed cochlea. Hear Res 124(1–2):1–16 9822898 10.1016/s0378-5955(98)00090-2

[7] Schaeper JJ, Liberman MC, Salditt T (2023) Imaging of excised cochleae by micro-CT: staining, liquid embedding, and image modalities. J Med Imaging (Bellingham) 10(5):053501 37753271 10.1117/1.JMI.10.5.053501 PMC10519431

[8] Walton LA et al (2015) Morphological characterisation of unstained and intact tissue micro-architecture by X-ray computed micro- and nano-tomography. Sci Rep 5:10074 25975937 10.1038/srep10074 PMC4650804

[9] Gulya J, ML, Poe DS (2010) Glasscock-Shambaugh surgery of the ear. In: Surgery of the ear, pp 619–642

[10] Brunschwig AS, Salt AN (1997) Fixation-induced shrinkage of Reissner’s membrane and its potential influence on the assessment of endolymph volume. Hear Res 114(1–2):62–68 9447919 10.1016/s0378-5955(97)00153-6

[11] Raufer S, Idoff C, Zosuls A, Marino G, Blanke N, Bigio IJ, O'Malley JT, Burgess BJ, Nadol JB, Guinan Jr. JJ, Nakajima HH (2020) Anatomy of the human osseous spiral lamina and cochlear partition bridge: relevance for cochlear partition motion. J Assoc Res Otolaryngol 21(2):171–182 32166603 10.1007/s10162-020-00748-1 PMC7270316

[12] Moore CW, Wilson TD, Rice CL (2017) Digital preservation of anatomical variation: 3D-modeling of embalmed and plastinated cadaveric specimens using uCT and MRI. Ann Anat 209:69–75 27777116 10.1016/j.aanat.2016.09.010

[13] Pujol S et al (2016) Using 3D modeling techniques to enhance teaching of difficult anatomical concepts. Acad Radiol 23(4):507–516 26897601 10.1016/j.acra.2015.12.012 PMC4808571

[14] Glueckert R et al (2018) Visualization of the membranous labyrinth and nerve fiber pathways in human and animal inner ears using microCT imaging. Front Neurosci 12:501 30108474 10.3389/fnins.2018.00501 PMC6079228

[15] van den Boogert T, Stephan Handschuh MVH, Glueckert R, Guinand N, Guyot J-P, Kingma H, Perez-Fornos A, Seppen B, Chacko LJ, Schrott-Fischer A, van de Berg R (2018) Optimization of 3D-visualization of micro-anatomical structures of the human inner ear in osmium tetroxide contrast enhanced micro-CT scans. Front Neuroanat 22(12):41 10.3389/fnana.2018.00041 PMC5972190 29872380

[16] Wright CG, Roland PS (2013) Vascular trauma during cochlear implantation: a contributor to residual hearing loss? Otol Neurotol 34(3):402–407 23222961 10.1097/MAO.0b013e318278509a

[17] Raufer S, Guinan JJ Jr, Nakajima HH (2019) Cochlear partition anatomy and motion in humans differ from the classic view of mammals. Proc Natl Acad Sci U S A 116(28):13977–13982 31235601 10.1073/pnas.1900787116 PMC6628837

